# Serum insulin-like growth factor-1 as a potential marker for MDD diagnosis, its clinical characteristics, and treatment efficacy validation: data from an open-label vortioxetine study

**DOI:** 10.1186/s12888-020-02636-7

**Published:** 2020-05-08

**Authors:** Oleg A. Levada, Alexandra S. Troyan, Irina Y. Pinchuk

**Affiliations:** 1 State Institution “Zaporizhzhia Medical Academy of Postgraduate Education Ministry of Health of Ukraine”, 20 Winter Boulevard, Zaporizhzhia, 69096 Ukraine; 2 Institute of Psychiatry of Taras Shevchenko National University of Kyiv, 64 Volodymirskaya Street, Kyiv, 01033 Ukraine

**Keywords:** IGF-1, Depression, Cognitions, Vortioxetine

## Abstract

**Background:**

IGF-1 is an essential neurotrophin produced peripherally and in the brain. Impairments in the brain IGF-1 concentrations might be responsible for some aspects of major depressive disorder (MDD) pathogenesis, whereas peripheral IGF-1 could have the marker value. We aimed: 1) to compare serum IGF-1 levels in MDD patients and healthy controls (HC); 2) to elucidate possible associations between changes in IGF-1 expression and crucial characteristics of the current depressive episode, MDD course; 3) to evaluate IGF-1 dynamics after 8 weeks` vortioxetine treatment.

**Methods:**

Seventy-eight MDD patients (according to DSM-5) and 47 HC were enrolled. Serum IGF-1, psychopathological (MADRS, CGI) and neuropsychological parameters (PDQ-5, RAVLT, TMT-B, DSST) were analyzed in all subjects at admission and 48 patients after 8 weeks` vortioxetine treatment. AUC-ROCs were calculated to determine if the value of serum IGF-1 could separate MDD patients from HC. Multiple regression models were performed to explore relationships between IGF-1 and depressive episode’s symptoms.

**Results:**

MDD patients had significantly higher serum IGF-1 levels than HC (228 (183–312) ng/ml vs 153 (129–186) ng/ml, *p* < 0.0001). IGF-1 had a good diagnostic value for predicting MDD in the whole sample with AUC of 0.820 (*p* < 0.0001). For a cutoff of 178.00 ng/ml, the sensitivity and specificity were 83 and 71%, respectively, and the number needed to misdiagnose was 5, indicating that only 1 of 5 tests give an invalid result. Among MADRS items, only reported sadness, inner tension, and concentration difficulties were significantly positively associated with serum IGF-1 concentrations. Vortioxetine treatment significantly attenuated IGF-1 levels and improved all psychopathological, neuropsychological parameters.

**Conclusions:**

Significant associations between IGF-1 levels and hypothymia, anxiety, and cognitive disturbances may indicate a pathogenic role of IGF-1 for the mentioned symptoms. We assume that the activity of the cerebral-hepatic axis increases in response to insufficient IGF-1 brain expression in MDD patients, whereas, vortioxetine treatment restores cerebral IGF-1 concentrations and, consequently, decreases its compensatory production by the liver.

**Trial registration:**

registered at ClinicalTrials.gov (NCT03187093). First posted on 14th June 2017.

## Background

Depression remains one of the crucial areas of scientific and applied psychiatric research over the past decades [1]. Such a situation is explained by the leading place of major depressive disorder (MDD) in the structure of psychiatric morbidity, as well as its social and economic consequences [2, 3]. MDD diagnosis is still based on clinical criteria [4], while modern treatment strategies are aimed at the deciphered biological pathways of the disorder [5]. Among them, special attention is paid to neuroplasticity [6–9]. Abnormal neural plasticity has been related to changes in the expression of neurotrophic factors [10]. Alterations in the signal pathways of neurotrophins can be considered as possible targets of therapy. At the same time, changes in their levels in body fluids can be used as biological markers of MDD diagnosis and treatment effectiveness.

Insulin-like growth factor-1 (IGF-1) is an essential neurotrophin that is produced both peripherally [11] and in the brain [12, 13]. It is involved in cell growth, differentiation, and maturation (through DNA synthesis and mitosis stimulation), in metabolic (i.e., glucose uptake and protein production) [13] and neuroplastic processes (synapses formation, neurotransmitters releasing, and exciting of neurons) [12]. Due to IGF-1 pleiotropic functions, it has been assumed that impairments in the IGF-1 system might be responsible for some aspects of MDD pathogenesis [13], as well as peripheral IGF-1 levels could have the marker value [14]. According to our recent review [14], the majority of studies demonstrate higher concentrations of peripheral IGF-1 in MDD patients compared to healthy controls (HC). Moreover, sufficient body of evidence suggests that antidepressant treatment decreases IGF-1 levels [14]. Nevertheless, it is still unclear whether the increased peripheral expression of the neurotrophin can be considered as a marker of the actual depressive episode (DE) itself, its structural domains (affective, cognitive or somatic disturbances), severity, clinical subtype or mentioned elevation is associated with the characteristics of the course of MDD (duration, number of episodes, etc.). Solving those questions could shed light on some aspects of MDD pathogenesis and contribute to improving the diagnosis of the disorder.

Taking into account the above arguments, our goals were: 1) to compare peripheral levels of IGF-1 in MDD patients and HC; 2) to elucidate possible associations between changes in IGF-1 expression and crucial characteristics of the current DE and MDD course; 3) to evaluate the dynamics of the neurotrophin levels after 8 weeks of treatment with vortioxetine.

## Methods

### Participants and procedures

This was a case-control study, which included 125 participants aged 18 to 65 years. Seventy-eight outpatients diagnosed with MDD according to DSM-5 criteria were recruited through Zaporizhzhia Regional Clinical Psychiatric Hospital, Ukraine. Eligibility criteria for the study inclusion were detailed elsewhere [15]. At study entry, all the patients received no actual antidepressant medication. Subjects were excluded if they had any other psychiatric diagnosis, high suicidal risk, substance dependence/abuse over the past year, significant neurological disorders, head trauma, unstable medical conditions, history of endocrine diseases, psychotic features in the current episode, high risk for a hypomanic switch. HC (*n* = 47) with no current psychiatric disorder were age-, sex-, and education-matched to MDD subjects. HC were excluded based on the use of medications and/or illicit drugs; the intake of alcohol within 48 h of the study visit; and the presence of an unstable medical condition, which could affect cognitive function.

**Fig. 1:**
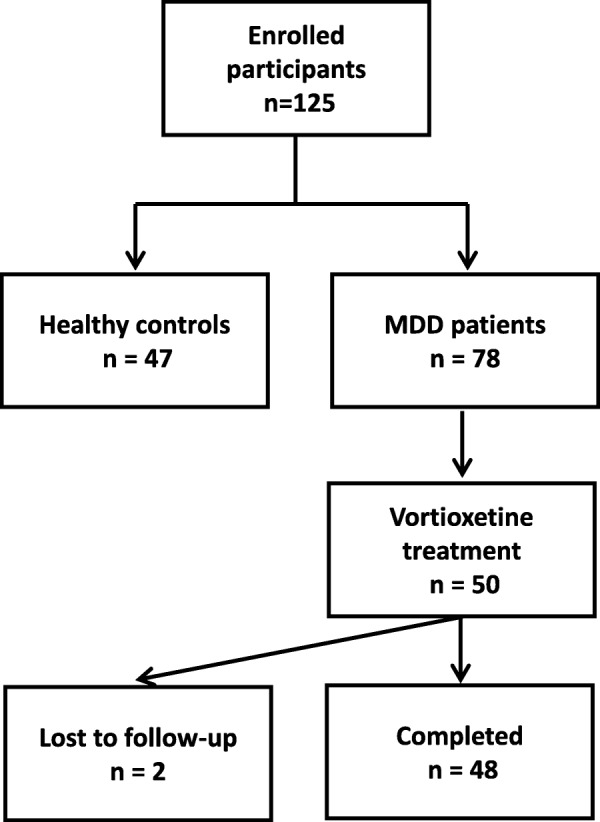
The flowchart of participant disposition

After confirmation of eligibility, participants provided written informed consent to take part in the study and attended a baseline visit to complete all evaluations (overall 125 subjects). The assessments were repeated in 48 patients after 8 weeks of treatment with flexibly-dosed vortioxetine 10–20 mg per day (two participants were lost for follow-up and not included). Only those patients receiving vortioxetine treatment were included in the analyses after treatment. The study flowchart is depicted in Fig. 1.

The study was approved by the local ethics committee and performed in accordance with the ethical standards laid down in the 1964 Declaration of Helsinki and its later amendments and registered at ClinicalTrials.gov (NCT03187093).

### Clinical assessments

Depression severity at baseline and changes after treatment were evaluated using MADRS [16] and Clinical Global Impression Severity (CGI-S) and Improvement (CGI-I) scales [17].

Subjective cognitive functioning was assessed using the Perceived Deficit Questionnaire-5 (PDQ-5), a 5-item self-report scale, which measures perceived difficulties of cognitive functioning. Each item ranges from 0 to 4, with higher scores reflecting greater severity [18].

### Neuropsychological assessments

To characterize the cognitive functioning, a selected battery that included neuropsychological tasks covering the most impaired cognitive domains in MDD was administered to all participants. The tests were managed using paper and pencil. The instruments included:

1. Rey Auditory Verbal Learning Test (RAVLT), to evaluate immediate verbal memory, retroactive and proactive interference effects, delayed recall and recognition [19];
2. Trail Making Test B (TMT-B), to assess processing speed, executive function, i.e., set shifting [19];
3. Digit Symbol Substitution Test (DSST), to evaluate processing speed, executive function, learning, memory, attention, and concentration [19]..

### IGF-1 measurements

In MDD patients and HC, IGF-1 concentration was measured in the morning under fasting conditions. Blood was drawn by a venous puncture between eight and eleven a.m. within the first 2 days after clinical assessments in 125 participants at baseline and 48 patients after 2 months of vortioxetine (10–20 mg per day) treatment. Blood was centrifuged at 3000 g for 10 min, and the serum was stored at − 20 °C until further processing. IGF-1 was measured using the chemiluminescence immunoassay Immulite 2500 (Siemens AG, Germany) and Human IGF-1 Quantikine ELISA Kit (R&D Systems Inc., Minneapolis, USA). As IGF-1 standard, the WHO IRR 87/518 was used, which offers a measurement range with the Immulite test from 20 to 1600 ng/ml.

### Statistical analysis

Data analyses were carried out with the statistical package SPSS for Windows, version 20.0 (SPSS Inc., USA). The results were given as median and interquartile range or means and SDs or percentages, depending on the data. The statistical significance of between-group comparisons was determined using non-parametric and parametric criteria when appropriate (Mann-Whitney test, Wilcoxon test, chi-squared test, T-test). To identify potential covariates for analyses for IGF-1 levels, preliminary correlational analyses were conducted between IGF-1 concentration and demographic and clinical variables using Spearman’s r_s_. After that, the areas under the receiver operating characteristic curves (AUC-ROC) were calculated to determine if the value of serum IGF-1 level could separate MDD participants from HC. A cutoff was derived from the ROC curve to yield empirical optimal sensitivity and specificity. The number needed to misdiagnose [20] was calculated for this cutoff. For ROC analyses, the whole sample was also divided into five groups according to age (18–24, 25–34, 35–44, 45–54, 55–65 years), as preliminary correlational analyses showed a significant negative association between age and IGF-1 concentrations. Afterward, multiple linear regression models were performed to explore potential relationships between IGF-1 levels and different symptoms of a DE (according to MADRS items). All four models included MADRS items and age, gender, level of education, and depression severity as covariates. The second model also included the number of the current DE; the third – included the duration of the current DE; the fourth – included both the number and length of the current DE. Besides, multiple linear regression models were performed to explore potential relationships between IGF-1 levels and the performance of each cognitive test, adjusted for the same variables (age, gender, education, depression severity, number and duration of the current DE). Lastly, we compared IGF-1 levels of 48 patients at baseline and after 8 weeks of treatment using Wilcoxon test for dependent samples. Significance was set at *p* < 0.05.

### Sample size determination

To determine the minimum of participants per group to compare IGF-1 levels, we used the following formula: 2SD^2^ (Z_α/2_ + Z_β_)^2^ / d^2^, where SD = standard deviation, Z_α/2_ = 1.96 at type 1 error of 5%, Z_β_ = 0.842 at 80% power, d = difference between mean values. A previous study revealed the following IGF-1 levels in MDD patients vs controls 157 ± 40 mg/ml and 120 ± 33 mg/ml, respectively [21]. Therefore, a minimum of 18 people per group had to be included. For regression analyses, we used Green’s rule, which requires a minimum of 104 + k subjects (where k is the number of predictors).

## Results

### Demographic and clinical characteristics of the sample

**Fig. 2:**
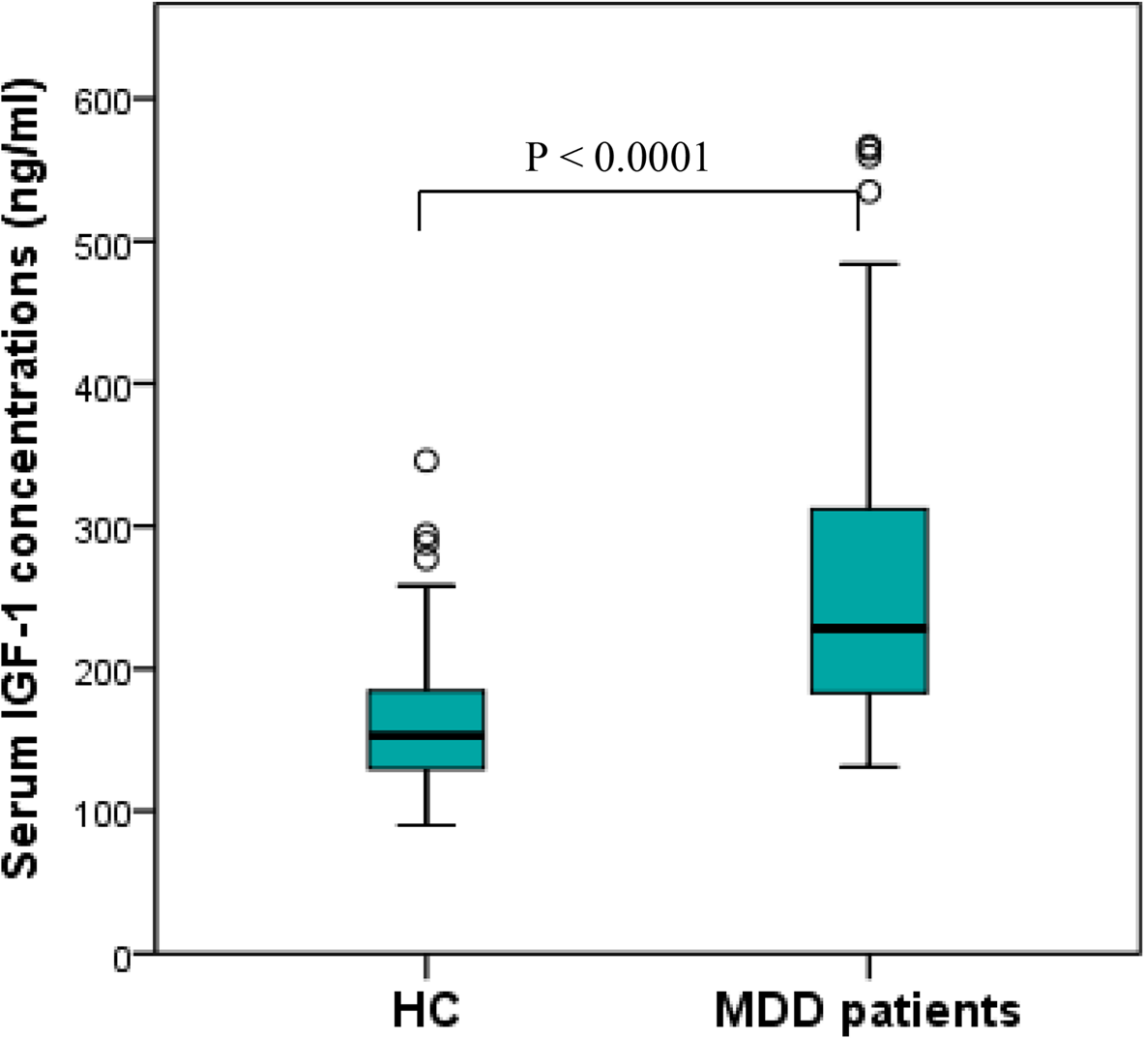
Serum IGF-1 levels in HC and MDD patients

The main demographic and clinical features and IGF-1 levels of the comparison groups are depicted in Table 1. Surveyed cohorts were comparable in age, gender, and level of education. Besides the expected statistical difference in MDD patients and HC on MADRS and CGI-S scales, sufficient distinction in neuropsychological test performance was found between the comparison groups. MDD participants were significantly worse (*p* < 0.0001) in executive functioning (DSST, TMT-B scores), processing speed (DSST, TMT-B scores), set shifting (TMT-B), and all parameters of verbal memory (RAVLT subtests). Regarding serum IGF-1, we detected significantly elevated levels of the neurotrophin in MDD patients as compared to controls (Fig. 2).

**Table 1:** Demographic, psychopathological, neuropsychological characteristics and serum IGF-1 levels in HC and MDD patients

	HC *n* = 47	MDD *n* = 78	*p*
***Demographic characteristics***			
Women, n (%)	27 (57.4%) ^a^	48 (61.5%)	0.65
Age, years^*^	37.8 (12.3) ^b^	38.2 (11.9)	0.84
Education, years^*^	15.2 (2.1) ^b^	14.5 (1.9)	0.60
***Clinical assessments***			
Psychopathological			
MADRS total score	2 (0–4) ^c^	29 (22–33)	**< 0.0001**
CGI-S score	1 (1–1) ^c^	4 (4–5)	**< 0.0001**
Patient-reported cognitive symptoms			
PDQ-5 total score	1 (0–2) ^c^	7 (5–11)	**< 0.0001**
Neuropsychological testing			
RAVLT immediate recall total score	64 (58–69) ^c^	49 (43–56)	**< 0.0001**
RAVLT proactive interference score	8 (6–9) ^c^	6 (5–7)	**< 0.0001**
RAVLT retroactive interference score	15 (13–15) ^c^	11 (9–12)	**< 0.0001**
RAVLT delayed recall score	15 (14–15) ^c^	11 (9–12)	**< 0.0001**
RAVLT delayed recognition score	15 (15–15) ^c^	14 (14–15)	**< 0.0001**
TMT-B (s)	57 (42–65) ^c^	77 (64–93)	**< 0.0001**
DSST number of correct symbols	62 (54–68) ^c^	50 (43–59)	**< 0.0001**
IGF-1 serum level (ng/ml)	153 (129–186) ^c^	228 (183–312)	**< 0.0001**

Data are presented as median (upper-lower quartile) unless otherwise stated; ^*^data are presented as means (SD); ^a^ Chi-square test, controls vs MDD patients; ^b^ T-test, controls vs MDD patients; ^c^ Mann–Whitney U-test, controls vs MDD patients.

### IGF-1 correlations with demographic, psychopathological and neuropsychological variables

Next, we performed a correlational analysis to determine possible associations between serum IGF-1 concentrations and demographic and clinical parameters in the whole sample (Table 2). We found a significant inverse correlation between serum IGF-1 levels and age and positive correlations between serum IGF-1 levels and the number, duration, and severity of the current DE. Additionally, we found that IGF-1 levels significantly correlated with the MADRS items and cognitive tests` scores.

**Table 2:** Spearman’s correlation coefficients between demographic, psychopathological, neuropsychological characteristics and serum IGF-1 levels in MDD patients and HC

Variables	r_s_
Age, years	−0.25**
Number of DE	0.31**
Duration of DE, weeks	0.42**
Education, years	−0.07
CGI-S score	0.51**
PDQ-5 total score	0.40**
MADRS total score	0.48**
Apparent sadness	0.45**
Reported sadness	0.50**
Inner tension	0.53**
Reduced sleep	0.30**
Reduced appetite	0.38**
Concentration difficulties	0.46**
Lassitude	0.36**
Inability to feel	0.47**
Pessimistic thoughts	0.53**
Suicidal thoughts	0.35**
RAVLT immediate recall total score	−0.34**
RAVLT proactive interference score	−0.23*
RAVLT retroactive interference score	−0.37**
RAVLT delayed recall score	−0.32**
RAVLT delayed recognition score	−0.05
TMT-B (s)	0.40**
DSST number of correct symbols	−0.21*

*p < 0.05; **p < 0.01

### IGF-1 levels for MDD diagnosis

**Fig. 3:**
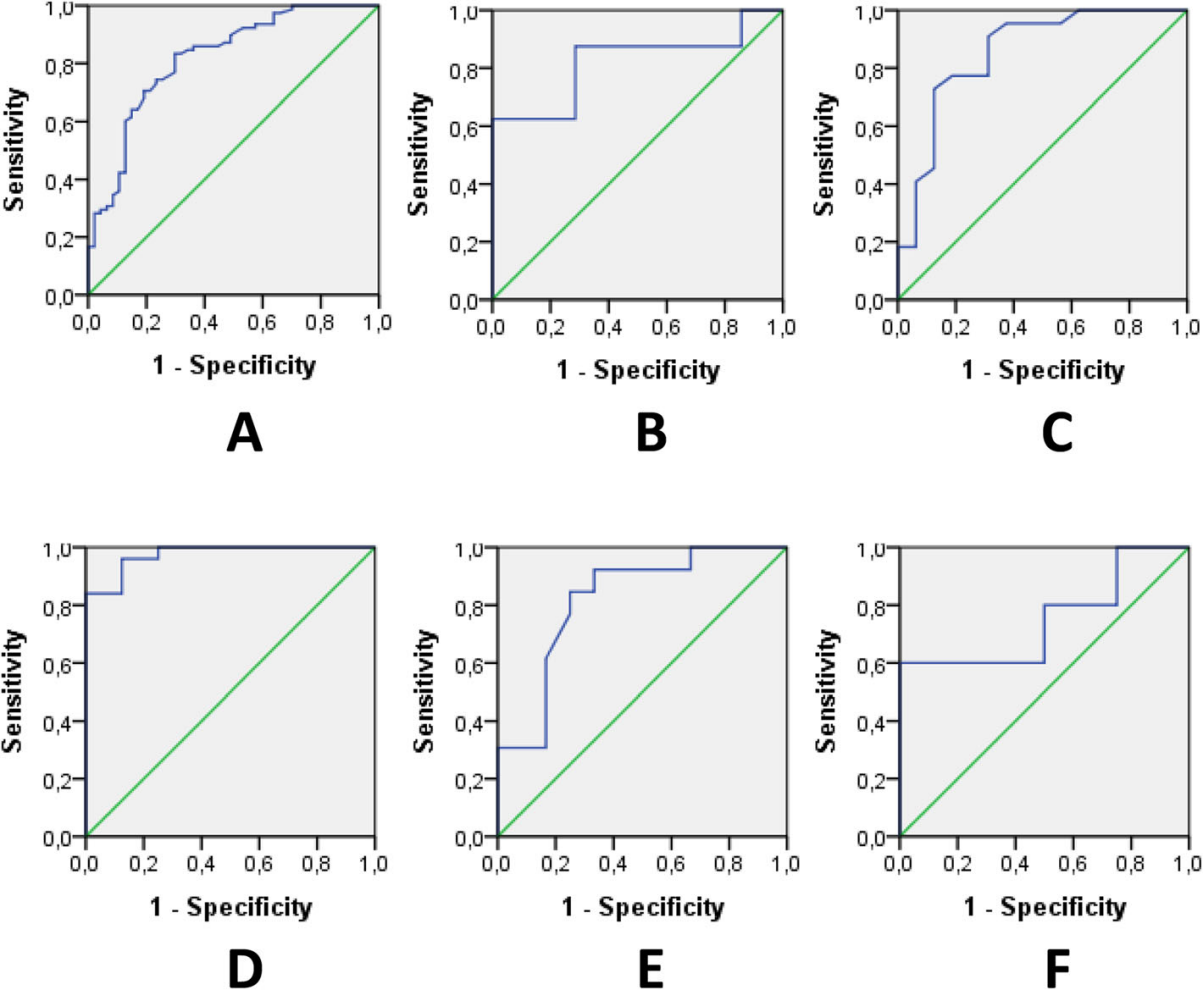
ROC curves for serum IGF-1 level and MDD status. **a** ROC curve to diagnose MDD in all age groups (18–65 y.o.) (*p* < 0.0001, AUC: 0.820; sensitivity: 83% and specificity: 71% for a cutoff of 178.00 ng/ml). **b** ROC curve to diagnose MDD in group 1 (18–24 y.o.) (*p* = 0.037, AUC: 0.821; sensitivity: 88% and specificity: 71% for a cutoff of 209.50 ng/ml). **c** ROC curve to diagnose MDD in group 2 (25–34 y.o.) (*p* < 0.0001, AUC: 0.855; sensitivity: 86% and specificity: 69% for a cutoff of 185.50 ng/ml). **d** ROC curve to diagnose MDD in group 3 (35–44 y.o.) (*p* < 0.0001, AUC: 0.975; sensitivity: 84% and specificity: 100% for a cutoff of 178 ng/ml). **e** ROC curve to diagnose MDD in group 4 (45–54 y.o.) (*p* = 0.007, AUC: 0.821; sensitivity: 85% and specificity: 75% for a cutoff of 176.00). **f** ROC curve to diagnose MDD in group 4 (55–65 y.o.) (*p* = 0.16, AUC: 0.750). AUC: area under the ROC curve; ROC: receiver-operating characteristic.

The discriminating ability of serum IGF-1 elevation to separate MDD participants from HC was determined by ROC analysis. Figure 3 represents the ROC curves for serum IGF-1 to diagnose MDD. IGF-1 had a good diagnostic value for predicting MDD in the whole sample with AUC of 0.820 (*p* < 0.0001). For a cutoff of 178.00 ng/ml, the sensitivity and specificity were 83 and 71%, respectively, and the number needed to misdiagnose was 5, indicating that only 1 of 5 tests give an invalid result. For AUC analyses, the whole sample was also divided into five age groups, as preliminary correlational analyses showed a significant negative association between age and IGF-1 concentrations. Age ranges for the groups were as follows: Group 1–18-24 years old (HC: *n* = 7, MDD: *n* = 8); Group 2–25-34 years old (HC: *n* = 16, MDD: *n* = 22); Group 3–35-44 years old (HC: n = 8, MDD: *n* = 25); Group 4–45-54 years old (HC: *n* = 12, MDD: *n* = 13); Group 5–55-65 years old (HC: *n* = 4, MDD: *n* = 10). We established that IGF-1 had a good diagnostic value for predicting MDD in groups 1, 2, and 4; and excellent discriminatory power in Group 3. On the contrary, serum IGF-1 level exhibited poor value for the diagnosis of MDD in persons older than 55 years (Group 5). The cutoff value of IGF-1 for groups 1–4 decreased with age. Thus, in Group 1 it was 210 ng/ml (sensitivity 88%; specificity 71%); in Group 2–186 ng/ml (sensitivity 86%; specificity 69%); in Group 3–178 ng/ml (sensitivity 84%; specificity 100%); in Group 4–176 ng/ml (sensitivity 85%; specificity 75%).

### Associations of IGF-1 concentrations with MDD symptoms

Multiple linear regression models were performed to explore potential relationships between IGF-1 levels and different symptoms of a DE (Table 3). All four models showed that among MADRS items, only reported sadness, inner tension, and concentration difficulties were significantly positively associated with serum IGF-1 concentrations when adjusted for other covariates, even though there was a prominent negative association between age and IGF-1 levels. Moreover, the magnitude of the association with hypothymia was the highest. No other significant correlations between serum IGF-1 and MDD features were observed.

**Table 3:** Multiple linear regression models of specific relationships between IGF-1(ng/ml) serum levels and MADRS symptoms (*n* = 125) ^a^

Variables	Model 1		Model 2		Model 3		Model 4	
	β (SE)	St. β	β (SE)	St. β	β (SE)	St. β	β (SE)	St. β
Age, years	**−1.81 (0.69)**	− 0.22*	**− 1.72 (0.69)**	− 0.21*	**− 1.80 (0.69)**	− 0.22*	**−1.72 (0.69)**	− 0.21*
Gender	27.10 (17.52)	0.13	25.81 (17.94)	0.13	26.37 **(**17.48)	0.13	24.29 (17.89)	0.12
Education, years	4.13 (3.98)	0.08	4.61 (3.97)	0.09	3.82 **(**3.98)	0.08	4.23 (3.96)	0.09
MADRS total score	−15.79 (8.45)	−2.26	− 13.78 (8.54)	−1.97	− 16.27 **(**8.44)	− 2.33	−14.05 (8.50)	− 2.01
MADRS items:								
*Apparent sadness*	−1.78 (16.54)	−0.03	−2.85 (16.45)	− 0.05	0.73 **(**16.61)	0.01	−0.24 (16.47)	− 0.004
*Reported sadness*	**46.96 (17.79)**	0.92*	**45.64 (17.73)**	0.89*	**45.90 (17.76)**	0.90*	**44.19** (**17.68)**	0.87*
*Inner tension*	**32.85 (10.09)**	0.55*	**30.62 (10.16)**	0.51*	**33.49 (10.07)**	0.56*	**31.03** (**10.12)**	0.51*
*Reduced sleep*	13.57 (10.31)	0.26	11.11 (10.33)	0.21	15.04 **(**10.34)	0.29	12.52 (10.33)	0.24
*Reduced appetite*	14.88 (12.08)	0.24	12.86 (12.05)	0.21	15.45 **(**12.06)	0.25	13.34 (11.99)	0.21
*Concentration difficulties*	**29.52 (11.68)**	0.51*	**27.43 (11.96)**	0.47*	**28.79 (11.67)**	0.50*	**26.11** (**11.94)**	0.45*
*Lassitude*	−5.56 (13.34)	−0.10	−8.99 (13.43)	−0.16	−4.97 **(**13.31)	− 0.09	− 8.70 (13.37)	− 0.16
*Inability to feel*	8.13 (13.35)	0.14	3.58 (13.72)	0.06	6.73 **(**13.36)	0.12	1.41 (13.74)	0.03
*Pessimistic thoughts*	**30.71 (13.29)**	0.51*	**27.72 (13.48)**	0.46*	**29.62 (13.28)**	0.49*	26.02 (13.47)	0.43
*Suicidal thoughts*	10.36 (12.21)	0.11	11.01 (12.13)	0.11	13.18 **(**12.38)	0.14	14.26 (12.29)	0.15
Number of DE		–	7.39 (6.17)	0.12		–	8.47 (6.19)	0.13
Duration of DE, weeks		–		–	0.225 **(**0.18)	0.1	0.25 (0.18)	0.12

^a^ IGF-1 level was entered as a dependent outcome and all MADRS symptoms together in the same model as independent variables, along with increasing confounder adjustment. β (SE) and standardized β are presented. Model 1 = adjusted for age, gender, education, and depression severity (according to MADRS); Model 2 = Model 1 + number of the DE; Model 3 = Model 1 + duration of the DE; Model 4 = Model 1 + number + duration of the DE; SE, standard error; *p < 0.05

### Association of IGF-1 and cognitive functioning in the whole sample

In addition, multiple linear regression models were performed to explore potential relationships between IGF-1 levels and the performance of cognitive tests by three separate linear regression analyses adjusted for relevant confounders (Table 4). Higher IGF-1 levels were significantly associated with the poor performance of all cognitive tests when adjusted for demographic covariates. Nevertheless, this association remained significant only between IGF-1 levels and DSST scores, when also adjusted for severity, number, and duration of the current DE (standardized β = − 0.21, *p* = 0.03).

**Table 4:** Multiple linear regression models of the associations between serum IGF-1 levels (ng/ml) and cognitive tests` performance (*n* = 125) ^a^

Variables	Model 1		Model 2		Model 3	
	β (SE)	St. β	β (SE)	St. β	β (SE)	St. β
RAVLT immediate recall	**−3.55 (0.87)**	− 0.35**	− 1.47 (1.10)	− 0.14	− 1.48 (1.08)	− 0.15
TMT-B	**1.29 (0.34)**	0.34**	0.63 (0.38)	0.17	0.52 (0.38)	0.14
DSST	**−2.93 (0.71)**	−0.38**	**−1.77 (0.76)**	− 0.23*	**− 1.62 (0.75)**	−0.21*

^a^ IGF-1 level was entered as a dependent outcome and each cognitive test score as independent variables, along with increasing confounder adjustment. β (SE) and standardized β are presented. Model 1 = adjusted for age, gender, and education, Model 2 = Model 1 + depression severity (according to MADRS), Model 3 = Model 2 + number + duration of DE; SE, standard error; *p < 0.05; **p < 0.0001

### The effect of antidepressant treatment on serum IGF-1 levels

After 8 weeks of vortioxetine treatment in 48 MDD patients, we revealed that the intake of the antidepressant significantly decreased serum IGF-1 concentrations and improved clinical parameters of patients (Table 5). Thus, we observed a significant decrease in depression severity and improvement of cognitive impairment (measured as subjectively as objectively).

**Table 5:** Changes in IGF-1 concentrations and different tests` scores after vortioxetine treatment

	At baseline *n* = 48	After treatment *n* = 48	Mean change	*p*
*Psychopathological symptoms*				
MADRS total score	29 (22–33)	6 (3–11)	20.8	**< 0.0001**
CGI-S score	4 (4–5)	1 (1–2)	2.3	**< 0.0001**
*Patient-reported cognitive symptoms*				
PDQ-5 total score	7 (4–10)	2 (1–3)	4.7	**< 0.0001**
*Performance-based cognition*				
RAVLT immediate recall	51 (43–55)	68 (64–72)	−15.9	**< 0.0001**
TMT-B (s)	75 (63–96)	47 (36–60)	30.1	**< 0.0001**
DSST	54 (42–61)	62 (51–71)	−9.7	**< 0.0001**
IGF-1 level (ng/ml)	236 (184–316)	170 (132–210)	89.0	**< 0.0001**

Data are presented as median (upper-lower quartile); *p* according to Wilcoxon test (paired samples)

## Discussion

In our study, we revealed that MDD patients had significantly higher serum levels of IGF-1 in comparison with HC. The most significant relationships were found between peripheral IGF-1 elevation and several DE symptoms – hypothymia, anxiety, and cognitive dysfunction. Eight weeks` vortioxetine treatment significantly attenuated serum IGF-1 concentrations and improved all psychopathological and neuropsychological parameters.

Regarding the peripheral IGF-1 increase in MDD patients, our findings are in line with some previous reports [21–26]. Nevertheless, the specific aspect of our study was that we obtained for the first time the cutoff values of serum IGF-1 to separate MDD patients from HC in different age groups, taking into account the proven fact of its decrease with age. Because of the small size of the age subgroups in our study, further work is needed to confirm these data.

The identification of peculiar DE symptoms having significant associations with IGF-1 levels was another distinctive feature of our study. Those were hypothymia, anxiety, and cognitive disturbances (particularly in executive functioning). This fact may indicate a pathogenic or compensatory role of IGF-1 for the development of the mentioned symptoms. The relationships between blood IGF-1 concentrations and cognitions in MDD have been previously studied only in the elderly population, and the results were contradictory to ours. Thus, Rueda et al. found a positive correlation with IGF-1 for cognition in women after adjustment for depression [27], a fact that was also reported in another study in healthy men [28]. Furthermore, decreased plasma IGF-1 levels were significantly associated with the decline of cognitive functioning and higher prevalence of depressive symptoms [29], whereas higher levels of IGF-1 were seen in those with improved cognition [30]. This inverse association found in elderly people may be explained by somatopause, which means that the secretion of IGF-1 decreases with advancing age in healthy adults [26, 31, 32].

The revealed discrepancies between IGF-1 increase in younger MDD patients and a decrease in older depressed individuals may indicate the unequal pathogenic role of the neurotrophin in these age-related cohorts. Reduction of cerebral and peripheral IGF-1 expression in the elderly may be directly related to the impairments in emotional and cognitive processing resulting in mood and neuropsychological symptoms. At the same time, the enhancement of the peripheral IGF-1 expression in young patients with MDD may be a compensatory mechanism in response to its brain synthesis decrease [25]. Also, the elevation of peripheral IGF-1 concentrations may be explained by the decreased cerebral bioavailability of the neurotrophin due to the reduced sensitivity of IGF-1 receptors under the neuroinflammatory stress [26]. Previously we have suggested that the activity of the cerebral-hepatic axis (GH/IGF-1 axis) increases in response to insufficient concentrations of this neurotrophin in the brain [33]. As a result, the production of IGF-1 in the liver elevates. When the production of IGF-1 in the brain is restored (i.e., after antidepressant treatment), the hepatic IGF-1 production and its blood concentration decline.

This suggestion is supported by the fact that IGF-1 concentrations in cerebrospinal fluid were found to be lower in MDD persons compared to HC and normalized after various antidepressant treatments irrespective of the class of medication, duration of therapy or response [34].

Data on a significant decrease of serum IGF-1 concentrations under vortioxetine treatment obtained in the study are consistent with the proposed concept and supported by several previous investigations. It was demonstrated that antidepressants – amitriptyline, doxepin, fluoxetine, paroxetine – led to a significant decline of peripheral IGF-1 levels [21, 35, 36]. For vortioxetine, such evidence was obtained for the first time to our knowledge. According to our hypothesis [33], we can assume that vortioxetine treatment restored cerebral IGF-1 concentrations and, consequently, decreased its compensatory production by the liver. Summarizing the evidence that a compensatory increase of peripheral IGF-1 in response to a deficiency in the brain may have pathogenetic effects for accelerating aging [33], we demonstrated that vortioxetine might have protective mechanisms against progeric effects, in particular telomerase attrition. As it was shown that IGF-1 induces the intracellular kinase-dependent pathways involved in proliferative processes and telomere attrition in various organs, which can be considered as the underlying mechanism of aging [37]. Nevertheless, the direct effect of vortioxetine on longevity needs further investigation.

## Conclusion

In conclusion, taken together, in the present study of the Ukrainian cohort, higher serum IGF-1 concentrations were associated with MDD status (in the age cohort of 18–54 years old), the severity of hypothymia, anxiety, and cognitive decline (predominantly executive dysfunction). This may indicate a pathogenic role of IGF-1 for the mentioned symptoms development. Treatment with vortioxetine substantially attenuated serum IGF-1 levels and improved all psychopathological and neuropsychological parameters. Further studies are needed to examine the relationships between IGF-1 and other neurotrophic factors in MDD patients and their influence on separate MDD symptoms.

## Data Availability

The datasets used and/or analyzed during the current study are available from the corresponding author on reasonable request.

## Abbreviations

AUC-ROC: Areas under the receiver operating characteristic curves
CGI-I: Clinical Global Impression Improvement
CGI-S: Clinical Global Impression Severity
DE: Depressive episode
DSM-5: Diagnostic and Statistical Manual of Mental Disorders, Fifth Edition
DSST: Digit Symbol Substitution Test
IGF-1: Insulin-like growth factor-1
HC: Healthy controls
MADRS: Montgomery-Asberg Depression Rating Scale
MDD: Major depressive disorder
NNM: Number Needed to Misdiagnose
PDQ-5: Perceived Deficit Questionnaire-5
RAVLT: Rey Auditory Verbal Learning Test
ROC: Receiver-operating characteristic
SD: Standard deviation
SPSS: Statistical Package for the Social Sciences
TMT-B: Trail Making Test B
